# Washed cell salvage in surgical patients: A review and meta-analysis of prospective randomized trials under PRISMA: Erratum

**DOI:** 10.1097/MD.0000000000010640

**Published:** 2018-04-27

In the article, “Washed cell salvage in surgical patients: A review and meta-analysis of prospective randomized trials under PRISMA”,^[1]^ which appeared in Volume 95, Issue 31 of *Medicine*, “/transplantation” was left out the main text and the supplemental digital content.

The last sentence of section 2.7 should appear as:

Subgroups were prospectively defined according to type of surgery (orthopedic, cardiac, vascular, multiple trauma/massive transfusion, cancer/transplantation, gynecology/obstetric, and pediatric) and age (pediatrics/adults) to determine whether effect sizes varied according to the type of surgery.

The first sentence of section 3.2 should appear as:

Of the 47 trials, 15 included orthopedic surgery,^[12,17,18,24–27,30,38,40,42,44,48,54,55]^ 21 cardiac surgery,^[9,14–16,20,22,23,28,29,32,33,35–37,41,43,46,47,50,51,53]^ 6 vascular surgery,^[13,19,31,45,49,52]^ 1 multiple trauma surgery,^[10]^ 2 cancer surgery/transplantation,^[21,39]^ and 2 pediatric surgery.^[11,34]^

In Table 1, the surgical discipline of the study Sankarankutty et al should state transplantation.

The heading for section 3.3.5 should be “Cancer surgery/transplantation.”

The first sentence of section 3.3.5 should be:

We found 2 studies^[21,39]^ that used cell salvage including 65 participants undergoing cancer surgery/transplantation.

The title for Supplemental Digital Content 6 should be Forest plot of cell salvage compared to no cell salvage in cancer surgery/transplantation.
